# Determinants of hydroxyurea use among doctors, nurses and sickle cell disease patients in Nigeria

**DOI:** 10.1371/journal.pone.0276639

**Published:** 2022-11-10

**Authors:** Hezekiah Alkali Isa, Uche Nnebe-Agumadu, Maxwell M. Nwegbu, Emmanuel C. Okocha, Reuben I. Chianumba, Biobele J. Brown, Samuel A. Asala, Emmanuel Peprah, Obiageli E. Nnodu

**Affiliations:** 1 Centre of Excellence for Sickle Cell Research and Training (CESRTA), University of Abuja, Abuja, Nigeria; 2 Department of Haematology, College of Health Sciences, University of Abuja, Abuja, Nigeria; 3 Department of Paediatrics, University of Abuja Teaching Hospital, Abuja, Nigeria; 4 Department of Chemical Pathology, University of Abuja Teaching Hospital, Abuja, Nigeria; 5 Department of Haematology, Nnamdi Azikiwe Teaching Hospital, Nnewi, Anambra State, Nigeria; 6 Department of Paediatrics, University College Hospital, Ibadan, Oyo State, Nigeria; 7 Department of Anatomical Sciences, College of Health Sciences, University of Abuja, Abuja, Nigeria; 8 Department of Social & Behavioral Sciences, New York University School of Global Public Health, New York, NY, United States of America; University of the West Indies Faculty of Medical Sciences Mona, JAMAICA

## Abstract

**Background:**

Hydroxyurea (HU) is an evidence-based therapy that is currently the most effective drug for sickle cell disease (SCD). HU is widely used in high-income countries with consequent reduction of morbidity and mortality. In Nigeria, HU is prescribed by physicians while nurses are mainly involved in counseling the patients to ensure adherence. The extent of utilization and the determinant factors have not been sufficiently evaluated in Nigeria.

**Objective:**

To assess the frequency of use of HU and factors affecting utilization among healthcare providers, patients, and caregivers for SCD.

**Methods:**

A questionnaire was administered online and in- person to assess the frequency of HU use and the factors that promote and limit its use. The data were analyzed by descriptive statistics using IBM SPSS software version 23 and the result was presented in frequency tables and percentages.

**Result:**

A total of 137 physicians, 137 nurses, and 237 patients/caregivers responded to the survey. The rate of prescription of HU by doctors in the past 6 months was 64 (46.7%), 43 (31.4%) nurses provided counseling and 36 (15.6%) patients were on HU. Among doctors, adequate knowledge (91.3%), clinical benefits and safety (94.8%), and inclusion of HU in management guidelines (86.9%) were motivators for prescribing it while inadequate knowledge (60.9%) and unawareness of treatment guidelines (68.6%) constituted barriers. Among nurses, reduction of crisis (91.6%) and safety (64.8%) were the major motivators while barriers were high cost (79.1%) and intensive monitoring (63.1%) of HU treatment. Among the patients, the major motivator was the reduction of crises (80.3%) while poor knowledge (93.2%), high cost of the drug (92.2%) while monitoring (91.2%), non-availability (87.7%) and side effects (83.9%) were the major barriers for the utilization of HU.

**Conclusion:**

HU prescription and utilization are still poor among healthcare providers and patients. Inadequate knowledge, non-availability and high cost of HU as well as unawareness of treatment guidelines constitute major barriers to prescription and utilization.

## Introduction

Sickle cell disease (SCD) is a hereditary disorder caused by a mutation in the gene responsible for the synthesis of the beta chain of haemoglobin [1]. The prevalence of sickle cell trait and the homozygous state in Nigeria (known to have the highest burden) is 25% and up to 2% respectively [2–4]. It is associated with many complications of varying severity and high mortality. Currently, few evidence-based pharmacological interventions are available for the treatment of individuals with SCD of which hydroxyurea (HU) is the most effective [5, 6].

Hydroxyurea was approved by the United States Food and Drug Administration (US-FDA) in 1998 for the treatment of SCD in adults [7]. Since then several studies have shown that HU is effective in reducing morbidity in SCD such as acute chest syndrome, vaso-occlusive crisis, stroke and blood transfusion requirements [8–14]. HU induces fetal hemoglobin production, mild myelosuppression, improves cellular hydration, reduces cellular adhesion and enhances the production of nitric oxide resulting in vasodilatation [7, 15]. In 2017 the approval was extended for use in pediatric patients 2 years of age and above by the US-FDA based on data from an open-label single-arm trial, by the European Sickle Cell Disease Cohort study (ESCORT HU, NCT02516579) [16, 17]. With this evidence HU is now widely used in developed countries and has greatly improved SCD outcomes. This is, however, not the case in Sub-Sahara Africa (SSA) including Nigeria due to fear of side effects, doubts about efficacy, lack of awareness of benefits and safety, non-availability and high cost among other barriers as reported in some studies across the region [18–24]. Nigeria through its Federal Ministry of Health, 2014 published guidelines for the management and control of SCD and recommended HU therapy for patients with severe clinical phenotypes [25] yet it has not significantly impacted HU usage as shown in recent studies. This study, therefore, aimed to assess HU utilization and its determinant factors among doctors, nurses and patients as indicated by the frequency of prescription, counseling of patients for HU treatment and uptake of HU respectively.

## Methodology

This study was a questionnaire-based survey with the target respondents being pediatricians, hematologists, public health physicians, resident doctors, general practitioners, nurses and SCD patients or their caregivers. Based on observations and experience from clinical practice and literature review, a structured questionnaire was developed to assess attitudes and practices of HU utilization and to reveal determinant factors that might underlie limited or non-utilization among healthcare providers, patients and caregivers. The questionnaire had 2 parts: demographics and frequency of use of HU (part 1) while barriers and motivators of use constituted part 2. A 4-point Likert scale (Strongly Agree, Agree, Disagree and Strongly disagree) was used to rate the response to each question on the barriers and facilitators to hydroxyurea usage. It took about 5 to 7 minutes to fill out the questionnaire. It was administered online using REDCap to which all professional platforms with large pools of the target respondents were given access via internet links. These platforms were the Nigerian Sickle Cell Support Society of Nigeria (SCSSN), Nigerian Society for Haematology and Blood Transfusion (NSHBT), Paediatric Association of Nigeria (PAN), Medical and Dental Association of Nigeria (MDCAN) National Association of Resident Doctors (NARD), National Association of Nigerian Nurses and Midwives (NANNM). Both the online and printed questionnaires were self-administered within a period of 6 months, from January to June 2021. Ethics approval was obtained from the National Health Research Ethics Committee of Nigeria (NHREC/01/01/2007-03/11/2020D), at the Federal Ministry of Health. The data collected was anonymous. Participation was voluntary for both online and in-person participants. Verbal consent was obtained from in-person participants and inferred for online respondents who completed the survey. The data were analyzed by descriptive statistics using IBM SPSS software version 23 and the result was presented in frequency tables and percentages.

### Participant selection

**Fig 1:**
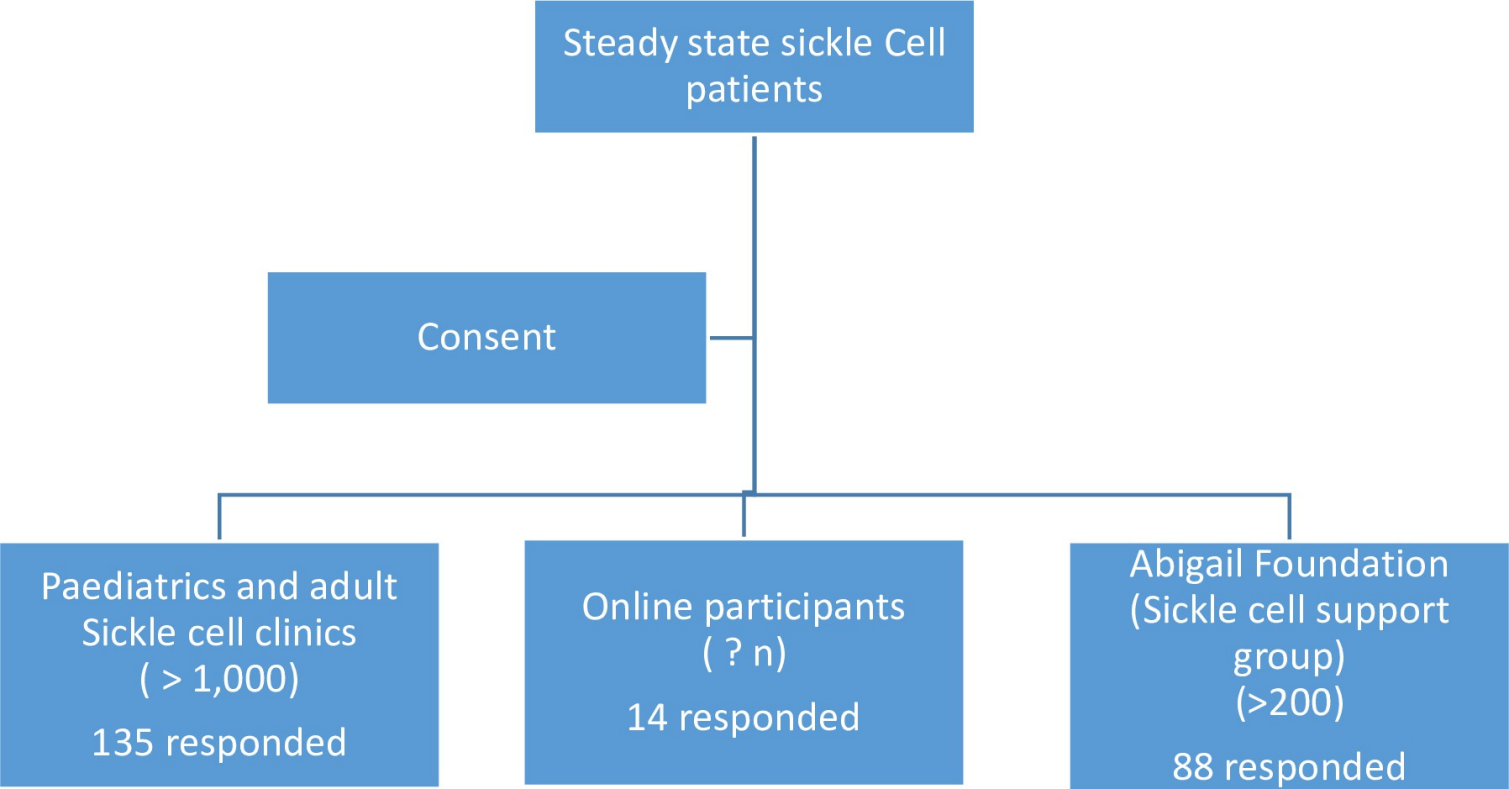
CONSORT diagram for patient participants (237 respondents).

We did not have any participant inclusion/exclusion criteria because all patients were attending SCD clinics and by definition had SCD. Our clinic has built significant rapport with our SCD patients and families over two decades. As such, the rapport translates to minimal refusal to participate in surveys. Thus, all consecutive patients that attend regular clinic visits defined as ‘steady state patients’ were administered the questionnaire over a 6-month period and most of them agreed to respond to it. In addition, the questionnaire was administered to sickle cell patients who are members of a sickle cell support group (Abigail foundation) during their regular meetings (Fig 1).

## Results

A total of 511 responses comprising 137 (26.8%) physicians, 137 (26.8%) nurses and 237 (46.4%) patients/caregivers were recorded.

### Frequency of utilization

In the last 6 months, 64 (46%) doctors prescribed HU, 43 (32.1%) nurses counseled patients on HU and 36(15.6%) patients were on HU.

### Motivators of HU Utilization

Table 3.
Table 5.

### Barriers

Table 4.
Table 6.

## Discussion

### Prescription of hydroxyurea

Hydroxyurea is mainly prescribed by doctors. Most of the doctors in this study are specialists, predominantly haematologists, paediatricians and their resident doctors who provide care to sickle cell patients in tertiary health care facilities. However, less than 50% prescribe HU and a majority of them prescribe it to less than 20% of their patients (Table 1). This is rather low considering that majority of the respondents have the highest level of training and provide care at the highest health care facilities where standard care is expected (Table 2). The major motivators for prescribing HU were adequate knowledge, clinical benefits and safety, cost-effectiveness and inclusion of HU in management guidelines (Table 3). This is expected because haematologists and paediatricians, should be conversant with HU, its efficacy and safety profile as well as its inclusion in the Nigerian National Guidelines for Management and Control of SCD. A similar study conducted among children with sickle cell disease in Tanzania identified adequate knowledge as a facilitator of HU usage among doctors and parents [18]. Inadequate knowledge, unawareness of treatment guidelines and high cost constituted barriers (Table 4). This corroborates a similar study on provider-related barriers to the use of HU [19] There is a need to create awareness of the existing Guidelines for management of SCD as well as training and retraining of doctors involved in the management of patients with SCD at all levels on the use and benefit of HU in SCD.

**Table 1:** Doctors versus percentage of their patients who are on HU.

Percentage of Patients on HU	Doctors (n = 64)
1–10%	21
11–20%	14
21–30%	11
31–40%	7
41–50%	5
>50%	5
Missing	1

**Table 2:** Doctors’ specialty.

Specialty	Frequency	Percentage
Haematologists	41	29.9
Paediatricians	35	25.6
Family Physicians	5	3.6
General Practitioners	3	2.2
Resident Doctors	30	21.9
Other Specialties	21	15.3
Missing	2	1.5
**Total**	137	100

**Table 3:** Motivators for HU prescription or counseling for HU among health care providers.

Motivators	Frequency (%)				
	Strongly agree	Agree	Disagree	Strongly disagree	Total
**DOCTORS**					
Good knowledge of HU	45 (39.1)	60 (52.2)	10 (8.7)	0 (0)	115 (100)
HU is cost-effective	21 (18.3)	63 (54.8)	30 (26.1)	1 (0.9)	115 (100)
HU has clinical benefits for patients	49 (4.6)	60 (52.2)	6 (5.2)	0(0)	115 (100)
The benefits of HU outweigh the side effects	36 (31.3)	73 (63.5)	6 (5.2)	0 (0)	115 (100)
Inclusion of HU in the treatment guideline	32 (27.8)	68 (59.1)	14 (12.2)	1 (0.9)	115 (100)
HU is available in the Hospital	12 (10.4)	44 (38.3)	48 (41.7)	11 (9.6)	115 (100)
HU is available in the community	9 (7.8)	39 (33.9)	55 (47.8)	12 (10.4)	115 (100)
**NURSES**					
HU reduces the frequency of Crises	50 (38.2)	70 (53.4)	7 (5.3)	4 (3.1)	131 (100)
Free HU	9 (6.9)	11 (8.5)	62 (47.7)	44 (33.8)	126 (100)
HU is affordable	11 (8.6)	46 (35.9)	58 (45.3)	13 (10.2)	128 (100)
Insurance cover cost and monitoring	8 (6.3)	22 (17.2)	68 (53.1)	29 (22.7)	127 (100)
HU has no serious side effects	15 (11.7)	68 (53.1)	38 (29.7)	6 (4.7)	127 (100)

**Table 4:** Barriers to HU prescription and counseling for HU among health care providers.

Barriers	Frequency				
	Strongly Agree	Agree	Disagree	Strongly Disagree	Total
**DOCTORS**					
Inadequate knowledge	11 (8.9)	64 (52.0)	38 (30.9)	10 (8.1)	123 (100)
There is no treatment guideline	13 (10.7)	45 (37.2)	51 (42.1)	12 (9.9)	121 (100)
Lack of awareness of treatment guidelines	24 (19.8)	59 (48.8)	30 (24.8)	8 (6.6)	121 (100)
Do not understand the treatment guideline	5 (4.1)	45 (37.2)	58 (47.9)	13 (10.7)	121 (100)
HU is expensive	13 (10.7)	57 (47.1)	42 (34.7)	9 (7.4)	121 (100)
Due to side effects	9 (7.4)	58 (47.9)	46 (38.0)	8 (6.6)	121 (100)
HU is not readily available	12 (9.9)	51 (42.1)	47 (38.8)	11 (9.1)	121 (100)
Burden of monitoring	14 (11)	40 (33.1)	53 (43.8)	13 (10.7)	120 (100)
**NURSES**					
Inadequate knowledge	11 (8.4)	20 (15.3)	61 (46.6)	37 (28.2)	130 (100)
Worry about side effects	25 (19.2)	53 (40.8)	41 (31,5)	10 (7.7)	129 (100)
HU is expensive for most patients	38 (29.5)	64 (49.6)	25 (19.4)	1 (0.8)	128 (100)
Monitoring increases the workload of Nurses	24 (18.5)	58 (44.6)	42 (32.3)	5 (3.8)	129 (100)
Patients have no knowledge of HU	43 (32.8)	66 (50.4)	13 (9.9)	7 (5.3)	131 (100)

### Motivators and barriers among nurses

Nurses play a very important role in counseling and educating patients in the clinics. As part of the patients’ workup for HU treatment, nurses educate and counsel them on HU and address questions or fears they may have about the treatment. This continues on every clinic visit during the general health talk given to the patients before the doctor’s consultation begins and helps with adherence. Only one-third of nurses counseled patients in the previous six months in this study. This low number may be a reflection of the number of sickle cell clinics in the country and the number of nurses attached to those clinics which is usually one or two nurses per clinic. The major facilitators for providing counseling are the nurses’ knowledge that HU reduces the frequency of crises and has no serious side effects (Table 3) while the high cost of HU and monitoring of treatment were the major barriers (Table 4). The provision of educational materials such as posters, flyers and video clips on SCD with emphasis on the benefits and safety of HU in the Clinics will make it easier for the few nurses who are attached to the very busy Clinics to educate and counsel the patients more effectively.

### Hydroxyurea usage, barriers and motivators among patients/caregivers

HU usage is still low in this study (15.6%) though better than frequencies reported in previous studies across the nation in the last decade (2014–2022) which ranged from 0 to 13.3% [19–22, 26] except one study which reported 47.5% in children [27]. The strongest motivator to HU usage was the reduction in the frequency of crisis (Table 5) while the high cost of drug, poor knowledge, non-availability and side effects, especially fear of infertility were the major barriers (Table 6). These findings are similar to those reported in three different studies in the country [18, 19, 21]. High cost of health care is generally a major challenge in Nigeria as the National Health Insurance coverage is still low and patients have to pay out-of-pocket for their drugs and other medical services [28]. It is important to note that the cost-effectiveness of HU in terms of reduction of crises, hospital admissions and other complications in the long term outweighs the burden of the high cost of HU [14, 29]. Moreover, HU is now relatively more affordable and available as it is now being produced locally [30]. Similarly, the fear of side effects may be due to inadequate information as HU has been shown to be a very safe drug in both children and adults when properly monitored [14, 18, 31]. Patients with SCD need continues education and counseling on the benefit and safety of HU. In addition, patients should be encouraged to subscribe to the National Health Insurance Scheme to be able to afford HU.

**Table 5:** Motivators for HU utilization among patients and care-givers.

Motivators	Frequency (%)				
	Strongly agree	Agree	Disagree	Strongly disagree	Total
**PATIENTS**					
Good Knowledge of HU	85 (40.7)	23 (11.0)	39 (18.7)	62 (29.7)	209 (100)
HU reduces the frequency of crisis	126 (64.0)	40 (20.3)	30 (15.2)	1 (0.5)	197 (100)
HU does not cause serious side effects	36 (18.6)	80 (41.2)	67 (34.5)	11 (5.7)	197 (100)
Insurance coverage for HU and Lab tests	15 (7.9)	16 (8.4)	115 (60.5)	44 (23.2)	190 (100)
HU is available	17 (8.6)	14 (7.1)	39 (19.8)	127 (64.5)	197 (100)
HU is affordable	13 (6.8)	8 (4.2)	36 (18.9)	134 (70.2)	191 (100)

**Table 6:** Barriers to HU utilization among patients and care-givers.

Barriers	Frequency				
	Strongly Agree	Agree	Disagree	Strongly Disagree	Total
**PATIENTS**					
I don’t have good knowledge about HU	163 (73.4)	44 (19.8)	2 (0.9)	3 (1.4)	222 (100)
HU is expensive	159 (82.4)	19 (9.8)	11 (5.7)	4 (2.1)	193 (100)
HU is not always available	158 (80.6)	14 (7.1)	20 (10.3)	4 (2.0)	196 (100)
Lab tests and hospital visits are expensive	93 (48.2)	83 (43.0)	13 (6.7)	4 (2.1)	193 (100)
Worry about Cancer	35 (18.1)	125 (65.8)	27 (14.2)	5 (2.6)	190 (100)
Worry about leg ulcer	28 (14.8)	87 (36.0	63 (33.3)	11 (5.8)	189 (100)
Worry about skin and nail changes	28 (14.7)	77 (40.5)	77 (40.5)	8 (4.3)	190 (100)
Worry about infertility	35 (18.2)	125 (65.1)	27 (14.1)	5 (2.6)	192 (100)
I use traditional medicine	11 (5.8)	4 (2.1)	24 (12.6)	152(79.6)	191 (100)
No need for HU because prayer works for me	8 (4.2)	4 (2.1)	27 (14.1)	152 (79.6)	191 (100)

## Conclusion

Hydroxyurea prescription and utilization are still low among healthcare providers and patients due to lack of knowledge, high cost/ non-availability of branded HU and concerns about side effects. There is a need for more health care providers and patient education and formulation of policies that will make HU more available and affordable.

### Limitation

Some limitations of this study include the self-administration of the questionnaire to patients and caregivers who may not understand and interpret questions uniformly, leading to information bias. The majority of the care providers who participated in the study were from tertiary institutions and may be more familiar with HU use. Their responses, therefore, may not represent that of health care providers generally. The strength of the study, however, lies in the large number of respondents that participated in the study.

## Supporting information

S1 Data (XLSX)

S2 Data (XLSX)

S3 Data (XLSX)
